# Characterizing the disability experience among adults living with HIV: a structural equation model using the HIV disability questionnaire (HDQ) within the HIV, health and rehabilitation survey

**DOI:** 10.1186/s12879-019-4203-0

**Published:** 2019-07-08

**Authors:** Kelly K. O’Brien, Steven Hanna, Patricia Solomon, Catherine Worthington, Francisco Ibáñez-Carrasco, Soo Chan Carusone, Stephanie Nixon, Brenda Merritt, Jacqueline Gahagan, Larry Baxter, Patriic Gayle, Greg Robinson, Rosalind Baltzer Turje, Stephen Tattle, Tammy Yates

**Affiliations:** 1 Department of Physical Therapy, University of Toronto, 500 University Avenue, Room 160, Toronto, Ontario Canada; 2 Rehabilitation Sciences Institute (RSI), University of Toronto, 500 University Avenue, Room 160, Toronto, Ontario Canada; 3 Institute of Health Policy, Management and Evaluation (IHPME), University of Toronto, Toronto, Ontario Canada; 4 Department of Health Research Methods, Evidence, and Impact, McMaster University, Hamilton, Ontario Canada; 5 School of Rehabilitation Science, McMaster University, Hamilton, Ontario Canada; 6 School of Public Health and Social Policy, University of Victoria, British Columbia, Canada; 7 Centre for Urban Health Solutions (CUHS), Li Ka Shing Knowledge Institute, St. Michael’s Hospital, Toronto, Ontario Canada; 8 Casey House, Toronto, Ontario Canada; 9 School of Health & Human Performance, Faculty of Health, Dalhousie University, Halifax, Nova Scotia Canada; 10 Community Member, Halifax, Nova Scotia Canada; 11 Gay Men’s Health Collective (GMHC), Three Flying Piglets, London, UK; 12 Community Member, Toronto, Ontario Canada; 13 Dr. Peter AIDS Foundation, Vancouver, British Columbia Canada; 14 Realize, formerly the Canadian Working Group on HIV and Rehabilitation, Toronto, Canada

**Keywords:** HIV/AIDS, Disability, Structural equation modeling, Aging, Rehabilitation, Uncertainty

## Abstract

**Background:**

People aging with HIV can experience a variety of health challenges associated with HIV and multimorbidity, referred to as ‘disability’. Our aim was to characterize the disability experience and examine relationships between dimensions of disability among adults living with HIV.

**Methods:**

We performed a structural equation modeling analysis with data from the Canadian web-based *HIV, Health and Rehabilitation Survey.* We measured disability using the HIV Disability Questionnaire (HDQ), a patient-reported outcome (69 items) that measures presence, severity and episodic features of disability across six domains: 1) physical symptoms, 2) cognitive symptoms, 3) mental-emotional health symptoms, 4) difficulties carrying out day-to-day activities, 5) uncertainty and worrying about the future, and 6) challenges to social inclusion. We used HDQ severity domain scores to represent disability dimensions and developed a structural model to assess relationships between disability dimensions using path analysis. We determined overall model fit with a Root Mean Square Error of Approximation (RMSEA) of < 0.05. We classified path coefficients of ≥ 0.2–0.5 as a medium (moderate) effect and > 0.5 a large (strong) effect. We used Mplus software for the analysis.

**Results:**

Of the 941 respondents, most (79%) were men, taking combination antiretroviral medications (90%) and living with two or more simultaneous health conditions (72%). Highest HDQ presence and severity scores were in the uncertainty domain. The measurement model had good overall fit (RMSEA= 0.04). Results from the structural model identified physical symptoms as a strong direct predictor of having difficulties carrying out day-to-day activities (standardized path coefficient: 0.54; *p* < 0.001) and moderate predictor of having mental-emotional health symptoms (0.24; *p* < 0.001) and uncertainty (0.36; *p* < 0.001). Uncertainty was a strong direct predictor of having mental-emotional health symptoms (0.53; p < 0.001) and moderate direct predictor of having challenges to social inclusion (0.38; *p* < 0.001). The relationship from physical and cognitive symptoms to challenges to social inclusion was mediated by uncertainty, mental-emotional health symptoms, and difficulties carrying out day-to-day activities (total indirect effect from physical: 0.22; from cognitive: 0.18; *p* < 0.001).

**Conclusions:**

Uncertainty is a principal dimension of disability experienced by adults with HIV. Findings provide a foundation for clinicians and researchers to conceptualize disability and identifying areas to target interventions.

**Electronic supplementary material:**

The online version of this article (10.1186/s12879-019-4203-0) contains supplementary material, which is available to authorized users.

## Background

People living with HIV taking combination antiretroviral therapy are reaching life expectancies parallel to HIV-negative populations [1, 2]. An estimated 30% of Canadians with HIV are 50 years or older and in 2014, an estimated 45% of people with HIV in the United States were aged ≥ 50 years, 27% aged ≥ 55 years, and 6% aged 65 and older [3, 4]. Similarly in the United Kingdom (UK), over half of people receiving HIV care will be 50 years or older by 2028 [5]. Similar trends have been projected in other countries with access to HIV treatment [6].

Even with improvements to survival, more individuals are living with a multitude of health-related concerns from HIV, aging and multimorbidity, including cardiovascular, liver and metabolic diseases, osteoporosis, and neurocognitive impairments [6–8]. From a rehabilitation science perspective, these health challenges, are described as ‘disability’ [9]. Disability can be episodic, exemplified by periods of illness and health that occurs over time [9]. The Episodic Disability Framework is a conceptual framework derived from, and validated with adults living with HIV. The Framework characterizes disability as a combination of symptoms and impairments (physical, cognitive, mental-emotional), difficulties carrying out daily activities, challenges with social inclusion and uncertainty or worrying about future health that can be experienced by an individual [9, 10]. Disability episodes can be amplified by extrinsic (e.g., experiencing stigma or absence of support) and intrinsic (e.g., aging, multimorbidity) contextual factors, adding further complications to the disability experience for people with HIV [11]. While the Episodic Disability Framework provided insights into the components that comprise disability, the way in which dimensions of disability relate to each other is unknown.

Measuring disability is essential for ascertaining the nature and extent of health challenges that comes from living with HIV and its associated multimorbidity, and the effectiveness of interventions for their ability to diminish or prevent disability. Authors of a systematic review explored disability among people with HIV in sub-Saharan Africa and found that 73% of included studies reported lower levels of function in HIV positive compared to HIV negative comparator groups, and a prevalence of 25% physical impairments in mobility and motor function among people with HIV [12]. Myezwa and colleagues (2009) described disability among people with HIV in the South African and Brazilian contexts, of which most commonly reported impairments included mental health function, fatigue and sleep health disorder [13]. Among adults hospitalized with HIV in South Africa, 100% experienced at least one or more impairment [14]. A provincial survey of people with HIV living in British Columbia, Canada found that the majority (more than 80%) of participants reported living with at least one impairment, activity limitation or social participation restriction; however, this study was conducted before the surge of multimorbidity associated with HIV and aging, likely underestimating the health complexities increasingly faced by older adults with HIV [15, 16]. Collectively these approaches to disability assessment in South Africa, Brazil, and Canada were guided by the International Classification of Functioning, Disability and Health (ICF) [17], which was not derived from the viewpoint of adults with HIV, and developed before the introduction of combination antiretroviral therapy; hence it may not fully encapsulate disability as experienced among those aging with HIV. Furthermore, the ICF suggests inter-relationships exist between all components of disability indicated with bidirectional arrows making it difficult to interpret for clinicians targeting interventions [17]. Better understanding the strength and direction of relationships among elements that comprise the broader concept of disability will help to identify precise areas for targeting interventions to most effectively reduce disability.

We examined relationships between components of disability using data from the Ontario HIV Treatment Network Cohort Study (OCS) using variables that resembled dimensions of disability within the Episodic Disability Framework, such as scores from symptom and quality of life questionnaires [18]. Results from this analysis indicated that physical and mental health challenges and difficulties carrying out daily activities either directly or indirectly predicted having challenges with social participation. However, we were unable to consider uncertainty in this analysis, a unique element of disability in the Episodic Disability Framework, because no questionnaires in the OCS captured this concept.

The HIV Disability Questionnaire (HDQ) is an HIV-specific patient-reported outcome measure of disability that was derived from the Episodic Disability Framework to assess disability presence, severity and episodic nature among adults living with HIV [19, 20]. The HDQ demonstrated sensibility, validity and reliability with people living with HIV in Canada, Ireland and the United States [21–24]. The HDQ is well suited for use by researchers, clinicians and community members to better understand the nature and extent of disability and the impact of aging with HIV with multimorbidity. Our aim was to characterize the disability experience and to examine the relationships between six dimensions of disability, specifically physical, cognitive, mental and emotional symptoms, difficulties carrying out day-to-day activities, uncertainty, and challenges to social inclusion among adults with HIV.

## Methods

We conducted a web-based cross-sectional survey with adults living with HIV in Canada to determine their profiles of disability. We then conducted a structural equation modeling (SEM) analysis to examine relationships among the six dimensions of disability as measured by the HDQ. We implemented a community-engaged approach to survey development, implementation, and data interpretation that included a team of researchers, clinicians, community members, and representatives from community-based organizations from Canada and the United Kingdom (UK), most of who are members of the Canada-International HIV and Rehabilitation Research Collaborative (CIHRRC) [25, 26]. We met face-to-face twice as a research and community collaborator team to discuss model interpretations and to determine the overall findings and implications for the HIV and clinical research community. We received ethics approval from Research Ethics Boards (REB) at the University of Toronto, McMaster University, Dalhousie University, and University of Victoria.

### Recruitment

We collaborated with 28 community-based organizations and HIV clinics to recruit adults living with HIV (≥ 18 years) in Canada [25]. Using a modified Dillman tailored design method, organizations administered four emails: 1) an initial email invitation comprised of an overview of the study and link to the questionnaire; 2) a thank you reminder email sent one week later; 3) another thank you reminder email sent approximately four weeks after the initial email; and 4) a final thank you reminder email sent one week later [27]. We enhanced our recruitment using electronic newsletters, website postings, and a promotional video, on-site posters and recruitment cards, and by word-of-mouth. Interested participants clicked on a link to access the survey instrument, which included an overview of the study, questions about eligibility criteria, and a question asking about whether individuals consent to participate in the study. We obtained written consent from participants by individuals answering ‘yes’ to the question ‘I agree to participate in this research study’. Participants were offered an electronic gift card worth $25 CAD for their participation.

### Survey instrument

The *HIV, Health and Rehabilitation Survey (HHRS)* instrument was developed, pre-tested and piloted by a group of researchers, people living with HIV, clinicians, and community collaborators across Canada. The instrument included a section on disability, utilization of rehabilitation services, concurrent health conditions, living (or coping) strategies, support, stigma, and personal characteristics, and took approximately 40 min to complete. We used Lime Survey software to administer the HHRS instrument [28]. In this manuscript, we specifically focused on disability and the characteristics of respondents living with HIV.

### Disability

The HIV Disability Questionnaire (HDQ) is a 69 item self-administered patient-reported outcome measure developed to describe the presence, severity and episodic nature of disability experienced by adults living with HIV. Comprised of six domains, the HDQ describes: physical symptoms and impairments (20 items), cognitive symptoms and impairments (3 items), mental-emotional symptoms and impairments (11 items), uncertainty and worrying about the future (14 items), difficulties carrying out day-to-day activities (9 items), and challenges to social inclusion (12 items) [20]. HDQ items were derived from categories in the Episodic Disability Framework, developed from perspectives of adults living with HIV [19, 20]. The HDQ possesses an ordinal response scale that ranges from ‘not at all’ ('0' response) having a challenge to ‘extreme challenge’ ('4' response). For every health challenge, participants are asked if that challenge changes in the past week (dichotomous scale: Yes or No). The HDQ is considered a sensible, reliable and valid instrument for measuring disability with adults living with HIV in Canada, Ireland and the United States [21–24].

#### Demographic and disease characteristics

The HHRS instrument included a series of items such as gender, age, year of HIV diagnosis, ethnicity, antiretroviral use, and geographical status. See Additional file 1 for the HHRS Survey instrument components relevant to this manuscript, including the demographic and clinical characteristics section. The HDQ items and domain structure has been published elsewhere [24].

### Analysis

We monitored view, participation and completion rates to the survey [29]. We downloaded data from Lime Survey [28] and analyzed missingness and floor and ceiling effects of HDQ items. We assessed distributions of HDQ item responses whereby floor and ceiling trends were defined as > 40% of responses at the ‘not at all’ (0) or ‘extreme’ (4) end of the scales, respectively. We analyzed demographic and disease characteristics descriptively with medians and interquartile ranges (IQR) (continuous variables) and frequencies and percent (categorical variables).

We computed HDQ scores for disability presence by adding the number of health challenges experienced; for severity scores, we summed individual item scores, and for episodic scores we summed the number of challenges that respondents identified as having fluctuated in the past week (episodic). All scores were linearly transformed into a score with a range of 0–100 with higher scores representing a greater presence, severity and episodic nature of disability.

**Fig. 1:**
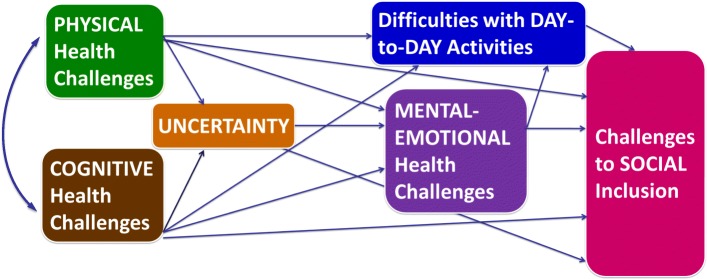
Hypothesized Structural Equation Model (SEM) of Relationships between Dimensions of Disability

We performed a structural equation modeling (SEM) analysis using HDQ severity scores to determine that strength of relationships between dimensions of disability as measured by the HDQ domain scores comprised of: 1) physical symptoms and impairments (20 items), 2) cognitive symptoms and impairments (3 items), 3) mental-emotional health symptoms and impairments (11 items), 4) difficulties with day-to-day activities (9 items), 5) uncertainty (14 items), and 6) challenges to social inclusion (12 items) [20]. In an earlier phase of work, we validated the HDQ with 361 people living with HIV in Ontario, Canada with confirmatory factor analysis. We established a measurement model and confirmed at the six HDQ domain scores represented disability dimensions [24]. Given our previous construct validity assessment confirmed that the HDQ items align with the six domain structure, we considered HDQ domain scores as representative of dimensions of disability [24]. Hence, for the purposes of this study, we used observed HDQ severity domain scores in our analysis. We constructed a structural model to examine direct and indirect (or mediating) effects between the disability dimensions using path analysis (Fig. 1). We derived our hypothesized model from our previous work examining relationships between different dimensions of disability [18].

We used the following a priori criteria to define goodness of model fit: Root Mean Square Error of Approximation (RMSEA) < 0.05, Tucker Lewis Index (TLI) ≥ 0.95, and Comparative Fit Index (CFI) ≥ 0.95 [30]. Of these, the RMSEA was considered our primary criterion for overall fit due to it commonly endorsed for goodness of fit for confirmatory factor analyses with SEM [30]. Although we reported the chi square statistic (χ^2^), due to the sensitivity of this statistic to larger sample sizes we did not take it into account as a factor for determining model fit in this study [31, 32].

We used Mplus 6.0 software [33] and maximum likelihood methods of estimation recommended for non-normal data [32, 34]. We performed mean imputation of HDQ missing responses in order to calculate subscale scores to maximize our sample size for the SEM analysis. We estimated unstandardized parameter estimates and reported standardized parameter estimates. Standardized parameter estimates were interpreted as the number of standard deviation increases above the mean in the outcome variable for every 1 standard deviation above the mean of the predictor variable. In this study, we arbitrarily defined standardized path coefficients of ≥ 0.2–0.5 as indicating a medium (or moderate) effect between the dimensions of disability and > 0.5 as having a large (or strong) effect.

Our hypothesized structural model had 20 unknown parameter estimates including variances of latent variables (*n* = 6) and parameter estimates between latent variables (*n* = 14). Guidelines suggest that for SEM analysis, 10 to 20 observations are required for each unknown parameter estimate [31, 32], hence our sample was well powered for this analysis.

## Results

Of the 4892 known invitations that were sent to adults living with HIV between October 2013–August 2014, 1850 (38%) accessed the survey link (view rate), of which 1477 (80%) started the questionnaire (participation rate), and 1171 (79%) finished the questionnaire (completion rate). We removed 230 responses (20%) because of high rates of missingness, nonsensical responses, and situations where we suspected multiple surveys responses. Hence there were 941 completed cases in the final dataset.

### Characteristics of participants

Of the 941 participants, the majority (79%) were men, who self-identified as gay, median age 48 years (IQR: 39, 54 years), taking antiretroviral medications (90%) and living with ≥ 2 concurrent health conditions (72%) (Table 1). The majority of respondents were from Ontario (71%). Thirty-seven percent of respondents were working either full or part time and 48% were living on an average gross personal income of <$20,000 CAD per year. The most frequent concurrent health conditions reported by > 20% of respondents included living with a mental health condition (e.g. anxiety or depression) (reported in 42% of the sample), muscle pain (33%), joint pain (30%), addiction (26%), and neurocognitive decline (22%).

**Table 1:** Characteristics of Respondents (*n* = 941)

Characteristic	Number (%)
*Gender*	
Men	740 (79%)
Women	159 (17%)
Transgender	19 (2%)
Two-spirited	15 (2%)
*Age*	
Age (median; IQR)	48 years (39–54 years)
50 years or older	405 (43%)
Born in Canada	692 (74%)
Living in a Metropolitan Area (defined as > 500,000 people)	636 (68%)
Living Alone	511 (54%)
Year of Diagnosis (median; IQR)	2000 (1992–2007)
Diagnosed Prior to 1996	323 (34%)
Taking Antiretroviral Medications	851 (90%)
Undetectable Viral Load (≤50 copies / mL)	572 (61%)
*Self-Reported General Health Status*	
Poor	28 (3%)
Fair	147 (16%)
Good	341 (36%)
Very Good	274 (29%)
Excellent	148 (16%)
*Comorbidities*	
Number of Concurrent Health Conditions (median; IQR)	3 (1–6)
Living with 2 or more concurrent health conditions in addition to HIV	518 (72%)

*IQR* interquartile range;

Not all values add to the total sample size of 941 due to missing responses

### Disability

There were low rates of missing responses (< 5%) across all HDQ presence and severity items. We performed mean imputation of HDQ scores to bring the dataset from 908 complete case responses to 941. For the episodic score, we did not conduct mean imputation as the missingness rate was 30% (284/941), likely attributed to respondents skipping episodic items if they did not consider themselves as having that challenge.

Thirty-four HDQ items possessed a floor effect with more than 40% of respondents rating these challenges as “0” or no disability. Floor effects (indicating ‘not at all’ disability) were most common to items about physical symptoms and impairments (*n* = 14), cognitive symptoms and impairments (*n* = 2), mental and emotional health symptoms and impairments (*n* = 3), difficulties carrying out day-to-day activities (*n* = 9) and challenges taking part in social and community life (*n* = 6). None of the HDQ items possessed a ceiling effect as defined as more than 40% of respondents rating challenges as “4” considered the highest disability severity.

HDQ disability scores were highest in the uncertainty domain as demonstrated by median presence (79; IQR: 57–93) and severity (38; IQR: 21–55) (Table 2). Among HDQ episodic scores (range 0–100), the highest median score that represented the domain with the most challenges that varied in the past week, was the physical symptoms and impairments domain (10; IQR: 0–35). The range of episodic scores for this domain ranged from 0 (not episodic at all) to 100 (all challenges varied in the past week). Common episodic health challenges included fatigue (30%; 226 respondents), feeling sad, down or depressed (26%; 229 respondents), diarrhea (26%; 229 respondents); headaches (27%; 228 respondents); aches and pains (26%; 221 respondents), trouble sleeping (26%; 234 respondents); and feeling anxious (25%; 223 respondents). The majority of respondents (80%; 749 respondents) rated themselves as having a ‘good day’ living with HIV when they completed the HDQ.

**Table 2:** HIV Disability Questionnaire (HDQ) Scores (n = 941)

Disability Dimension	Median Presence Score (IQR)	Median Severity Score (IQR)	Median Episodic Score (IQR) [Range; n]
Physical Symptoms and Impairments	55 (35–75)	21 (11–32)	10 (0–35) [0–100; 657]*
Cognitive Symptoms and Impairments	67 (0–100)	17 (0–33)	0 (0–33) [0–100; 866]
Mental-Emotional Symptoms and Impairments	73 (36–95)	27 (14–48)	0 (0–36) [0–100; 808]
Uncertainty	79 (57–93)*	38 (21–55)*	0 (0–21) [0–100; 811]
Difficulties Carrying out Day-to-Day Activities	33 (0–78)	11 (0–31)	0 (0–22) [0–100; 823]
Challenges to Social Inclusion	67 (33–92)	29 (12–48)	0 (0–8) [0–100; 798]
HDQ Total Score	62 (41–78)	25 (13–40)	6 (0–26) [0–99]

*IQR* interquartile range; *HDQ* HIV Disability Questionnaire; HDQ scores range from 0 to 100 with higher scores indicating a greater presence, severity and episodic nature of disability; *Boldface indicates highest scores across HDQ domains for each scale; n for episodic scores specified for each HDQ domain because imputation not performed due to increased missing responses for these items

### Relationships between dimensions of disability

**Fig. 2:**
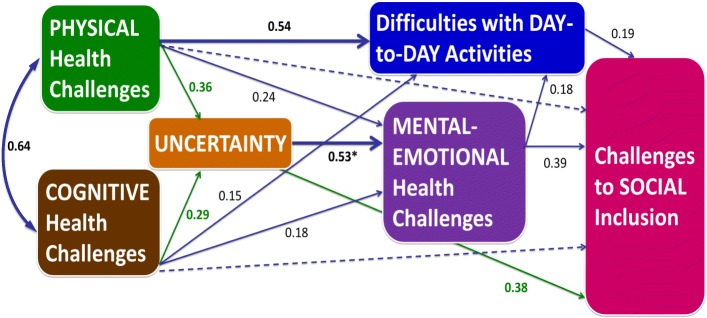
Final Structural Equation Model (SEM) of Relationships between Dimensions of Disability. Goodness of Fit Statistics: Comparative Fit Indices (CFI): 1.00; Tucker Lewis Index (TLI): 0.993; Root Mean Square of Error of Approximation (RMSEA): 0.04. Numbers indicate standardized path coefficients; the magnitude of the relationship (Range is 0–1); Solid lines indicate direct effect; Dashed lines indicate non-significant relationship; Curved lines indicate correlations. Bolded path coefficients indicate strong (> 0.50) predictive relationships; Green arrows indicate strongest mediating relationship. *Example Interpretation: An increase in uncertainty 1 standard deviation above the mean predicts a 0.53 standard deviation increase above the mean in mental-emotional health challenges (while controlling for physical, cognitive, and social challenges)

The six HDQ domain scores represented six dimensions of disability as indicated by overall good model fit (RMSEA = 0.04). Results from the path analysis in the structural model showed that physical symptoms and impairments strongly predicted difficulties with day-to-day activities (standardized path coefficient: 0.54; *p* < 0.001) and moderately predicted mental-emotional health symptoms (0.24; *p* < 0.001) and uncertainty (0.36; p < 0.001). Uncertainty strongly directly predicted mental-emotional health symptoms (0.53; p < 0.001) and moderately directly predicted challenges to social inclusion (0.38; p < 0.001). In other words, an increase in uncertainty 1 standard deviation above the mean predicted a 0.53 standard deviation increase above the mean in mental-emotional health challenges (while controlling for physical, cognitive, and social challenges). Uncertainty had a moderate direct influence on social inclusion (0.38; p < 0.001) and largest indirect influence on social inclusion (0.53 × 0.39 = 0.21; p < 0.001) through effects on mental-emotional health challenges, even after adjusting for physical and cognitive health challenges (Fig. 2). After taking uncertainty and mental-emotional health challenges into account, physical and cognitive health challenges had no direct effects or minimal indirect effects (< 0.20) on social inclusion through difficulties with day-to-day activities, which itself had a small effect on social inclusion (0.19; p < 0.001). See Table 3 for a summary of the model parameter estimates.

**Table 3:** Structural Equation Model Parameter Estimates for Determining Relationships among Dimensions of Disability

Relationships among Dimensions of Disability	Unstandardized Parameter Estimates (95% CI)	Standardized Parameter Estimates (95% CI)
Challenges to Social Inclusion		
on Difficulties Carrying out Day-to-Day Activities	0.23 (0.16, 0.30)*	0.19 (0.13–0.25)*
on Mental-Emotional Symptoms and Impairments	0.37 (0.30, 0.43)*	0.39 (0.32–0.45)*
on Uncertainty	0.36 (0.30, 0.42)*	0.38 (0.32–0.44)*
on Cognitive Symptoms and Impairments	−0.02 (−0.08, 0.04)	−0.02 (−0.07–0.04)
on Physical Symptoms and Impairments	0.04 (−0.05, 0.14)	0.03 (− 0.03–0.09)
Mental – Emotional Health Challenges		
on Uncertainty	0.53 (0.48, 0.58)*	0.53 (0.48, 0.58)*
on Cognitive Symptoms and Impairments	0.20 (0.14, 0.27)*	0.18 (0.12, 0.24)*
on Physical Symptoms and Impairments	0.37 (0.29, 0.46)*	0.24 (0.18, 0.30)*
Difficulties Carrying Out Day-to-Day Activities		
on Mental-Emotional Symptoms and Impairments	0.14 (0.08, 0.19)*	0.18 (0.11–0.24)*
on Cognitive Symptoms and Impairments	0.13 (0.07, 0.19)*	0.15 (0.08, 0.22)*
on Physical Symptoms and Impairments	0.65 (0.57, 0.73)*	0.54 (0.47, 0.60)*
Uncertainty		
on Cognitive Symptoms and Impairments	0.32 (0.24, 0.41)*	0.29 (0.21–0.37)*
on Physical Symptoms and Impairments	0.56 (0.44, 0.68)*	0.36 (0.29–0.43)*
Physical Symptoms and Impairments		
Correlated with Cognitive Symptoms and Impairments	217.5 (187.92, 246.38)*	0.64 (0.60–0.68)*

Structural Equation Model Results: Relationships represented by HDQ Severity Scores. Chi-square statistic (χ^2^): 2.51; *p* value: 0.11; Degrees of freedom: 1; Comparative Fit Index (CFI): 1.00 (ideal is ≥ 0.95); Tucker-Lewis Index (TLI): 0.99 (ideal is ≥ 0.95)Root Mean Square Error of Approximation (RMSEA): 0.04 (good fit represented by < 0.05); *significant p < 0.001; Note: ‘on’ refers to relationships where parameter estimates represent regression coefficients; ‘correlated with’ indicates correlation coefficient

## Discussion

Results illustrate the first known profile of disability experienced by adults with HIV using the HDQ, a unique HIV-specific patient-reported disability questionnaire. Uncertainty and worrying about the future with HIV was a key dimension of disability for participants in this study. Uncertainty as conceptualized by the Episodic Disability Framework, and measured by the HDQ refers to the extent an individual worries about when a potential episode of illness might occur, the severity and potential outcomes of an illness, and the impact worrying about future health with HIV may have on life decisions such as returning to work, purchasing a home, initiating new relationships, or starting a family [9]. Uncertainty may be experienced with difficulty identifying sources of health challenges, particularly given new and emerging health challenges may be attributed to a combination of HIV, chronic inflammation, long-term antiretroviral use, multimorbidity, and aging. This can pose challenges when trying to identify interventions to address the source of disability. Authors who qualitatively examined the disability experience among 49 older men and women living with HIV identified uncertainty as a vital feature of episodic disability. They attributed uncertainty to unknown causes of health-related challenges, lack of or newly emerging knowledge and skills about HIV and aging among health providers, financial and housing insecurity, and concern about who will meet their caregiving needs as they get older with HIV [35, 36].

However, some argue uncertainty can be beneficial for adopting strategies of hope and optimism and stimulate considerations for advanced care planning with a chronic terminal illness [37–39]. Uncertainty can incite ways for individuals to adopt attitudes of hope and optimism and ‘live in the present’ rather than allowing the threat of a terminal illness to overburden and weigh on the mind [37, 38]. Perrett and Biley (2013) used qualitative approaches with adults living with HIV to establish theory focused on ‘negotiating uncertainty’ comprised of seven categories ranging from ‘anticipating hopelessness’ to ‘regaining optimism’ [39]. Solomon and colleagues (2018) identified six themes to define successful aging with HIV, some of which directly refer to strategies for living with uncertainty such as: acceptance, remaining positive, maintaining social connections and support with others, taking responsibility and control of health, keeping up a healthy lifestyle, and taking part in meaningful activities [40].

Mental and emotional health challenges was a prominent mediator between uncertainty to challenges to social inclusion, and the second most severe dimension of disability. This aligns with anxiety and depression (mental health) that was reported as the most common concurrent health condition by 42% of respondents. The prevalence of depression and anxiety can be as high as 72 and 82% among people with HIV, respectively [41]. As social isolation is a concern for adults aging with HIV [36, 42], it is critical for health providers to consider approaches for addressing mental health challenges and uncertainty.

Episodic health challenges among respondents most commonly occurred in the physical and mental and emotional health domains including fatigue (30%), feeling sad, down or depressed (26%), diarrhea (26%); headaches (27%); aches and pains (26%), trouble sleeping (26%); and feeling anxious (25%). These results are similar to the most prevalent, distressing and burdensome symptoms measured with a group of adults with HIV in the United States [43]. Fatigue is correlated with depression and is more prevalent among people with HIV compared to the general population [41, 44]. Our sample similarly indicated fatigue and depression were among the highest reported and severely experienced health challenges. Physical and mental health challenges identified as episodic in this study (fatigue, feeling sad, down or depressed, feeling anxious, headaches, aches and pains, and trouble sleeping) highlight specific symptoms and impairments in which health professionals can tailor interventions to help mitigate the sometimes fluctuating nature of disability for adults with HIV.

Uncertainty emerged as an important direct and indirect predictor of experiencing mental-emotional health challenges and having challenges participating in society (social inclusion) with HIV. These findings build on our previous SEM work [18] with the OCS study [45], where “physical symptoms and impairments, mental-emotional health symptoms impairments, and difficulties with day-to-day activities directly or indirectly predicted challenges to social inclusion for adults living with HIV” [18]. Data in this prior study were derived from instruments that measured constructs that resembled disability (e.g. quality of life, symptom indices) but did include measures of uncertainty [18]. Hence, this study builds on this work to include and highlight the critical importance of uncertainty in the disability experience.

Interestingly, physical and cognitive health challenges had minimal to no effect on social inclusion after taking uncertainty and mental-emotional health challenges into account. Earlier work identified the prominent role of mental health in mediating the link between physical symptoms to challenges to social inclusion [18]. Oberje and colleagues (2015) examined relationships between components of HRQL and subjective well-being (defined as life satisfaction, affective experiences and mood) and found mental health was one of the strongest direct predictors of self-reported well-being opposed to physical health, which was only weakly associated [46]. Nevertheless, physical symptoms and impairments should not be ignored [47]. Collectively, results suggest the interplay between all dimensions of disability, and in particular, the prominence of uncertainty and mental and emotional health in determining social inclusion for adults living with HIV.

Results from this study possess theoretical implications by broadening our understanding of disability as articulated in the Episodic Disability Framework [9]. Our original development of the Framework derived from qualitative inquiry suggested relationships exist between disability dimensions, but the nature, extent and direction of those relationships were unknown. Hence, this work helps to further our understanding of disability by highlighting uncertainty as a key dimension and its predictive influence on mental health and social inclusion. Our analysis focused on examining relationships of dimensions of disability that comprise our core concept of interest as defined by the Episodic Disability Framework [9]. We did not consider the influence of contextual factors on dimensions of disability [11]. Positive coping and social support can positively influence satisfaction with life and work, motivation, and gratification (defined as global function), whereas stigma can negatively influence global function among adults living with HIV [48]. Socioeconomic factors (education, health insurance, employment) and lifestyle factors (smoking, physical activity) also may influence self-reported disability [49]. Future research should examine the influence that extrinsic (support, stigma) and intrinsic factors (age, sex, gender, sexual orientation, multimorbidity, living strategies) have on dimensions of disability in order to identify potential interventions to mitigate or prevent disability.

Respondents in this study were living with multiple coexisting health conditions. Respondents had a median of three health conditions, and 72% of the sample were living with ≥ 2 simultaneous health conditions. With the most common simultaneous health conditions of mental health (42%), muscle pain (33%), and joint pain (30%), we expect that disability reported by respondents was attributed to HIV, multimorbidity, and associated treatments, although items on the HDQ do not ascertain the source of health challenges. Disentangling the source of disability is important for future work. Almost half of the HHRS sample was ≥ 50 years of age. With the increase in life expectancy, more people aging with HIV will be living with increasing multimorbidy and associated health challenges [6, 8]. Greater burden of comorbidity is associated with lower physical HRQL [50]. For instance, older adults with HIV reported lower physical HRQL; but greater comorbidity burden was not associated with poorer mental health quality of life scores across age groups [50]. These results suggest that despite greater comorbidity with age, older adults may have adopted resilience and self-management strategies to alleviate mental and emotional health challenges living with HIV [51].

Frailty is an increasingly important consequence of HIV and aging associated with greater morbidity and mortality compared with HIV-negative controls [52–55]. Only 6% of the HHRS sample reported living with frailty as a concurrent health condition, which may be due to participants’ lack of knowledge that they may be living with this condition, and the nature of frailty assessment and classification [56]. Erlandson and colleagues (2014) identified an association between frailty and difficulty with instrumental activities of daily living, such as housekeeping and transportation, suggesting that difficulties with day-to-day activities may become increasingly prominent as adults age with HIV and frailty [53]. There is an increasing need for interprofessional approaches involving infectious disease, gerontology and rehabilitation disciplines to work collectively in addressing the complexity of health challenges among those aging with HIV [57].

### Implications for clinical practice

Results from this work highlight the importance for clinicians and community members to consider uncertainty and its impact on mental health, function and social inclusion for adults aging with HIV. Collectively, health providers have a role in targeting efforts to address uncertainty by discussing concerns, values and priorities that may pertain to worrying about one’s future health aging with HIV. This dialogue can help to provide support to those struggling with uncertainty by acknowledging the reality of living with a chronic and sometimes unpredictable episodic illness [37].

### Implications for future research

This study provides the groundwork for further disability research in the context of HIV. Our analytical focus in this study was on the core concept of disability, however future modeling may determine the influence of extrinsic (e.g. social support, stigma) and intrinsic contextual factors (e.g. living (or coping) strategies) on dimensions of disability [11]. Our findings will help empirically to identify areas for targeting interventions to mitigate disability experienced by adults with HIV. Forthcoming work also should consider adjusting for personal characteristics such as age, sex, gender, time since diagnosis and type of comorbidities to examine whether they moderate the relationships between different dimensions of disability.

We measured disability using a self-reported outcome measure opposed to performance-based measures of function and disability focused on assessing activities of daily living tasks and motor skills [58]. The HDQ enabled us to capture self-reported performance on function and mobility as well as perceptions of mental and emotional health challenges and uncertainty, which are difficult domains to ascertain using performance based measurement approaches. While not specific to HIV, evidence supports the assertion that self-reported and performance-based assessments of activities of daily living measure related, but distinct aspects of ability [59]. Nevertheless, considering both performance-based (functional ability) and perception-based assessments of disability are important for predicting complex health challenges in the ‘real world’ context. Future research may consider a combination of objective and self-reported approaches for measuring disability in the context of aging with HIV.

### Limitations

This study was comprised of a convenience sample of adults with HIV recruited primarily from community service organizations across Canada, comprised largely of gay men living in urban geographical areas. While the largest proportion of respondents were from Ontario, which similarly reflects the province with the highest HIV prevalence rates in Canada [3], our study was not bilingual, which limited the inclusion of French-speaking residents of Quebec and Newfoundland and Labrador; thus findings do not characterize disability among the broader population of adults with HIV in Canada. Future work is underway to examine the influence of gender on the disability experience. The meaning of the HDQ scores should be considered prudently given the interpretability (or clinical importance) of the scores is unclear. The episodic nature of health challenges may be overestimated due to the large rate of missing responses; however, this did not impact our SEM analysis which used severity scores. Our mean imputation of missing HDQ scores was a limitation that hence may have resulted in an overestimation of the precision of scores [60, 61]. Out of the 941 cases included in the analysis, 908 (96%) had complete HDQ data. We used mean imputation at the time of HDQ scoring to recover the subscale HDQ scores prior to the SEM analysis to bring the dataset from 908 complete case responses (96%) to 941. Severity items we used to compute the subscale scores that were used in the analysis had few missing responses, ranging from 1 missing response (0.1%) for HDQ51 (I have trouble climbing stairs) to 33 missing responses (3.5%) for HDQ19 (I have problems with my hearing). Nevertheless, our mean imputation remains a limitation; future SEM work should consider full implementation maximum likelihood (FIML) methods to ‘preserve’ characteristics of the data, so that parameter estimates possess minimal bias, meaning they are derived from a mean and variance as close as possible to that of the true population [60]. Next, interpretations of the strengths of standardized path coefficients may vary. Our a priori interpretations of the strengths of standardized path coefficients as predictors of disability in this study were arbitrary, and consistent with values used in previous latent variable modeling of disability in the context of HIV [18]. Finally, because of the cross-sectional nature of our analysis, we were not able to draw inferences regarding causality. We hypothesized our direct relationships in our structural model based on our previous work [18] and supported by literature that suggests physical health predicts mental health [62]. However, in reality the relationships between these dimensions are likely more complex. Exploration of disability using longitudinal analytical approaches may help to determine causation and the impact of interventions in addressing disability over time.

## Conclusions

Uncertainty is a fundamental important feature of disability experienced by adults living with HIV. Among the six dimensions of disability, uncertainty was a direct and indirect predictor of experiencing mental-emotional symptoms and impairments and challenges to social inclusion in the context of HIV. Results provide the groundwork for enhancing our understanding of the disability experience and can be used to highlight areas of where to target interventions that can assist to prevent or mitigate disability and enhance health outcomes for adults aging with HIV.

## Additional file

Additional file 1: HIV Health and Rehabilitation Survey (HHRS) Instrument components relevant to this manuscript, including the demographic and clinical characteristics section. The HIV Disability Questionnaire has been published elsewhere: O’Brien KK, Solomon P, Bayoumi AM. Measuring Disability Experienced by Adults Living with HIV: Assessing Construct Validity of the HIV Disability Questionnaire using Confirmatory Factor Analysis. *BMJ Open.* September 1, 2014. 2014; **4**:e005456 doi:https://doi.org/10.1136/bmjopen-2014-005456. Available at: https://bmjopen.bmj.com/content/4/8/e005456 (PDF 237 kb)

## Data Availability

Data may be available by request to the corresponding author on reasonable request.

## Abbreviations

FIML: Full implementation maximum likelihood
HDQ: HIV Disability Questionnaire
HIV: Human immunodeficiency virus
HRQL: Health-related quality of life
IQR: Interquartile range
