# Supplementary material for: Characterizing the disability experience among adults living with HIV: a structural equation model using the HIV disability questionnaire (HDQ) within the HIV, health and rehabilitation survey
Source: BMC Infect Dis. 2019 Jul 8;19:594. doi: 10.1186/s12879-019-4203-0 (PMC6615082; doi:10.1186/s12879-019-4203-0)
Supplement: Supplementary file 1 — HIV Health and Rehabilitation Survey (HHRS) Instrument components relevant to this manuscript, including the demographic and clinical characteristics section. The HIV Disability Questionnaire has been published elsewhere: O’Brien KK, Solomon P, Bayoumi AM. Measuring Disability Experienced by Adults Living with HIV: Assessing Construct Validity of the HIV Disability Questionnaire using Confirmatory Factor Analysis. BMJ Open. September 1, 2014. 2014; 4:e005456 doi:https://doi.org/10.1136/bmjopen-2014-005456. Available at: https://bmjopen.bmj.com/content/4/8/e005456 (PDF 237 kb) [file 12879_2019_4203_MOESM1_ESM.pdf]

Additional File 1

Characterizing the Disability Experience among Adults Living with HIV: A Structural Equation Model Using the HIV Disability Questionnaire (HDQ) within the HIV, Health and Rehabilitation Survey

HIV, Health and Rehabilitation Survey

The aim of this survey is to describe the health-related challenges experienced by adults living with HIV, identify other health conditions they may be living with, and the rehabilitation services and living strategies that they use to manage their health challenges.

Supplemental File 1 –includes HHRS sections specific to this manuscript including Section A (Demographic and clinical characteristics). Section B (HDQ) is published elsewhere

[Section A1] About You

This section asks some background questions about you.

A01. How did you first find out about this survey? I heard about the survey from…..

- ❑ Positive Living Society of British Columbia (formerly known as, British Columbia Persons with AIDS Society [BCPWA], (Vancouver, British Columbia) (1)
- ❑ Dr. Peter Centre / Dr. Peter AIDS Foundation (Vancouver, British Columbia) (2)
- ❑ Nine Circles Community Health Centre (Winnipeg, Manitoba) (3)
- ❑ AIDS Coalition of Nova Scotia (Halifax, Nova Scotia) (4)
- ❑ Casey House (Toronto, Ontario) (5)
- ❑ Canadian Working Group on HIV and Rehabilitation (CWGHR) (6)
- ❑ Toronto People with AIDS Foundation [PWA] (Toronto, Ontario) (7)
- ❑ HIV Edmonton (HIV Network of Edmonton Society) (Edmonton, Alberta) (8)
- ❑ AIDS Committee of Toronto [ACT] (Toronto, Ontario) (9)
- ❑ The Teresa Group (Toronto, Ontario) (10)
- ❑ Northern AIDS Connection Society [NACS] (Truro, Nova Scotia) (11)
- ❑ AIDS Committee of Newfoundland and Labrador [ACNL] (St. John's Newfoundland & Labrador) (12)
- ❑ AIDS Saint John (Saint John, New Brunswick) (13)
- ❑ Positive Women's Network [PWN] (Vancouver, British Columbia) (14)
- ❑ A friend notified me about the survey (15)
- ❑ Other – Please describe below (16)

Additional File 1

Characterizing the Disability Experience among Adults Living with HIV: A Structural Equation Model Using the HIV Disability Questionnaire (HDQ) within the HIV, Health and Rehabilitation Survey

A01a. You indicated that you found out about this survey from an “Other” source. Please describe: _____ [conditional if A01a is Other]

A02. What province or territory do you currently live in?

- ❑ Newfoundland and Labrador (1)
- ❑ Prince Edward Island (2)
- ❑ Nova Scotia (3)
- ❑ New Brunswick (4)
- ❑ Quebec (5)
- ❑ Ontario (6)
- ❑ Manitoba (7)
- ❑ Saskatchewan (8)
- ❑ Alberta (9)
- ❑ British Columbia (10)
- ❑ Yukon (11)
- ❑ Northwest Territories (12)
- ❑ Nunavut (13)

A03. Which of the following best describes the **geographic** area where you currently live?

- ❑ Metropolitan (population 500,000 or more people) (1)
- ❑ Urban (population 100,000 or more and less than 500,000 people) (2)
- ❑ Rural (population less than 100, 000 people) (3)

A04. As of today, what is your age (in years)? _________

A05. What best describes your gender?

- ❑ Woman (0)
- ❑ Man (1)
- ❑ Trans: Man to woman (2)
- ❑ Trans: Woman to man (3)
- ❑ Two-spirited (4)
- ❑ Inter-sexed (5)
- ❑ Other – Please describe below (6)

A05a. You indicated your gender as “Other”. Please describe: ________ [conditional if A05 is Other]

A06. What best describes your sexual orientation?

- ❑ Heterosexual/Straight (0)
- ❑ Lesbian (1)
- ❑ Gay (2)
- ❑ Bisexual (3)
- ❑ Queer (4)
- ❑ Questioning (5)
- ❑ Two-Spirit (6)
- ❑ Asexual (7)
- ❑ Other – Please describe below (8)

A06a. You indicated your sexual orientation as “Other”. Please describe: _______ [conditional if A06 is Other]

A07. What is your current marital or partnership status?

- ❑ Married, living common-law with partner or living in a committed relationship (1)
- ❑ Separated or Divorced (2)
- ❑ Single (3)
- ❑ Widowed (4)
- ❑ Don’t know (88)

A08. Do you have any children?

- ❑ Yes (1)
- ❑ No (0)

A09. Please describe who you currently live with. Check ‘Yes’ or ’No’ for each of the following:

	Yes	No
a) I live alone.	☐	☐
b) I live with my child/children (dependent).	☐	☐
c) I live with my adult child/children.	☐	☐
d) I live with my partner or spouse.	☐	☐
e) I live with my siblings.	☐	☐
f) I live with my parents.	☐	☐
g) I live with my extended family relations.	☐	☐
h) I live with unrelated people (e.g. with friends or roommates).	☐	☐

A10. In what year were you diagnosed with HIV? _______________

A11. Are you currently taking anti-retroviral (HIV) medications?

- ❑ Yes (1)
- ❑ No (0)

A11a. In what year did you start taking anti-retroviral (HIV) medications? _____

A12. Do you have a primary health care provider (e.g. family doctor, nurse practitioner, primary care nurse)?

- ❑ Yes (1)
- ❑ No (0)

A13. Are you currently receiving care from an HIV doctor or attending a clinic for HIV care?

- ❑ Yes (1)
- ❑ No (0)

A14. Are you currently receiving care from a naturopathic HIV doctor?

- ❑ Yes (1)
- ❑ No (0)

A15. In general, would you say your health is:

- ❑ Excellent (1)
- ❑ Very Good (2)
- ❑ Good (3)
- ❑ Fair (4)
- ❑ Poor (5)

A16. Compared to one year ago, how would you rate your health in general now?

- ❑ Much better now than one year ago (1)
- ❑ Somewhat better now than one year ago (2)
- ❑ About the same as one year ago (3)
- ❑ Somewhat worse now than one year ago (4)
- ❑ Much worse now than one year ago (5)

Additional File 1

Characterizing the Disability Experience among Adults Living with HIV: A Structural Equation Model Using the HIV Disability Questionnaire (HDQ) within the HIV, Health and Rehabilitation Survey

A17. Listed below are some health conditions that individuals may live with in addition to HIV. For each one, check 'Yes' if you are <u>currently</u> living with the following conditions and 'No if you are not. We are interested in knowing if you are living with these conditions, even if you are managing them with medications.

**Condition**	**Yes (1)**	**No (0)**
a) Addiction (e.g. alcohol, drugs, or gambling)	☐	☐
b) Arrhythmia (irregular heart beat)	☐	☐
c) Asthma	☐	☐
d) Bone and joint disorder (e.g. osteonecrosis (interrupted blood supply to bone); osteopenia or osteoporosis (decreased bone density) or osteoarthritis) or osteoarthritis (degenerative joint disease caused by loss of joint cartilage)	☐	☐
e) Cancer (Please describe below)	☐	☐
f) Cardiovascular disease (e.g. coronary artery disease, heart attack, angina, stroke)	☐	☐
g) Chronic Obstructive Pulmonary Disease (COPD) (e.g. chronic bronchitis, emphysema)	☐	☐
h) Dementia	☐	☐
i) HIV Associated Neurocognitive Disorder (HAND)	☐	☐
j) Diabetes	☐	☐
k) Elevated triglycerides (triglycerides are a type of fat found in the blood. Elevated levels are considered equal to or greater than 2.3 mmol/L.[1]) *Check 'yes' if you are managing your triglycerides with medication.*	☐	☐
l) Eye disorder (e.g. glaucoma, macular degeneration)	☐	☐
m) Fibromyalgia	☐	☐
n) Frailty (a complex condition commonly associated with age that can include a combination of physical, cognitive, functional and social circumstances that place someone at risk of harmful health outcomes)	☐	☐
o) Hepatitis B co-infection	☐	☐
p) Hepatitis C co-infection	☐	☐
q) High blood pressure (defined as having a systolic blood pressure equal to or greater than 140 mm Hg and a diastolic blood pressure equal to or greater than 90 mm Hg (140/90 mm Hg).[2]) *Check 'yes' if you are managing high blood pressure with medication.*	☐	☐

Condition	Yes (1)	No (0)
r) High cholesterol (elevated levels of cholesterol in the blood (hypercholesterolemia) (defined as having higher concentrations of low-density lipoproteins (>3.4mmol/L) and lower concentration of functional high-density lipoproteins (<1-1.3mmol/L) levels in the blood.[3]) *Check 'yes' if you are managing high cholesterol with medication.*	☐	☐
s) HPV (human papillomavirus)	☐	☐
t) Joint pain (arthritis)	☐	☐
u) Kidney disease	☐	☐
v) Liver disease	☐	☐
w) Mental health condition (e.g. depression, anxiety)	☐	☐
x) Multiple Sclerosis	☐	☐
y) Muscle pain	☐	☐
z) Neurocognitive decline (e.g. memory loss, confusion, trouble thinking clearly or solving day-to-day problems)	☐	☐
aa) Obesity (when excess body fat has accumulated to the extent that it has harmful effects on your health; or when body mass index (which compares weight and height) is greater than 30kg/m^2)	☐	☐
bb) Osteonecrosis (e.g. interrupted blood supply to bone)	☐	☐
cc) Osteopenia or osteoporosis (e.g. decreased bone density)	☐	☐
dd) Osteoarthritis (e.g. degenerative joint disease caused by loss of joint cartilage)	☐	☐
ee) Parkinson's Disease (or Parkinsonism)	☐	☐
ff) Peripheral artery disease	☐	☐
gg) Peripheral neuropathy	☐	☐
hh) Rheumatoid Arthritis (e.g. chronic inflammation of the joints)	☐	☐
ii) Stomach Ulcers	☐	☐
jj) Thrombosis (blood clots)	☐	☐
kk) Tuberculosis (TB)	☐	☐
ll) Other (Please describe below)	☐	☐

A17e. You indicated that you are currently living with Cancer. Please describe the type of Cancer: ___ [conditional if A17e is 'yes']

A17ll. You indicated that you are currently living with an Other health condition (Other #1). Please describe: ____ [conditional if A17ll is 'yes']

A17a-A17ll. You indicated that you are currently living with [SUBSITUTE HEALTH CONDITION from A17a-A17ll, NAME ONLY]. Were you diagnosed with this health condition **before** you were diagnosed with HIV? [conditional if any of the A17a to A17ll is 'yes']

- ❑ Yes (1)
- ❑ No (0)

The definitions of the health conditions were derived from these sources:

1) Mayo Foundation for Medical Education and Research. High Cholesterol - Triglycerides: Why do they matter?. 2013. Available from: http://www.mayoclinic.com/health/triglycerides/CL00015.
2) Heart and Stroke Foundation. Getting your blood pressure in check. 2012. Available from: http://www.heartandstroke.com/site/c.ikIQLcMWJtE/b.3484023/k.2174/Heart_disease__High_blood_pressure.htm.
3) Mayo Foundation for Medical Education and Research. High Cholesterol - Cholesterol Levels: What numbers should you aim for? 2013. Available from: http://www.mayoclinic.com/health/cholesterol-levels/CL00001.

[Section B] Health Challenges

This section asks about the presence, severity and episodic nature of health-related challenges that you might experience living with HIV and any other health conditions.

HIV Disability Questionnaire

HIV Disability Questionnaire (HDQ)

The HIV Disability Questionnaire is under copyright license © 2017, Kelly O'Brien. Patty Solomon, Ahmed Bayoumi, McMaster University; University of Toronto. All rights reserved.

To request permission to use the HDQ go to: https://milo.mcmaster.ca/Copyrighted%20Works/HDQ. Any modification, adaptation, translation or derivative works of the HDQ are prohibited.

The HDQ items and domain structure has been published elsewhere:

O'Brien KK, Solomon P, Bayoumi AM. Measuring Disability Experienced by Adults Living with HIV: Assessing Construct Validity of the HIV Disability Questionnaire using Confirmatory Factor Analysis. *BMJ Open*. September 1, 2014. 2014;4:e005456 doi:10.1136/bmjopen-2014-005456
Available at: https://bmjopen.bmj.com/content/4/8/e005456

[Section A2] More About You

This last section of the survey asks a few more questions about you.

This information will help us describe who completed the survey as a group and help us understand how factors like employment status may relate to health service use.

D01. Were you born in Canada?

- ❑ Yes (1)
- ❑ No (0)

D01a. What year did you come to Canada to live? ______________
[conditional if D01 is 'no']

D01b. Are you: [conditional if D01 is 'no']

- ❑ A Canadian citizen (1)
- ❑ A landed or permanent resident (2)
- ❑ A refugee (3)
- ❑ Here with temporary work papers (4)
- ❑ Here with humanitarian and compassionate approval (5)
- ❑ A student (6)
- ❑ A visitor (7)
- ❑ Other – Please describe below (8)
- ❑ Prefer not to say (55)
- ❑ Don't Know (88)

D01c. You indicated "Other" in the previous question (D01b). Please describe: ______
[conditional if D01b is 'other']

D02. Do you identify yourself with a particular ethnic or cultural group?

- ❑ Yes (1)
- ❑ No (0)

D03. Please check the option that **best** describes your ethnocultural background.

- ❑ First Nations (1)
- ❑ Metis (2)
- ❑ Inuit (3)
- ❑ Caucasian-White (English language) (4)
- ❑ Caucasian-White (French language) (5)
- ❑ Caucasian-White (non-English speaking or French speaking) (6)
- ❑ South Asian (e.g. East Indian, Pakistani, Punjabi, Sri-Lankan) (7)
- ❑ Chinese (8)
- ❑ Black or African (9)

- ❑ African-Canadian (10)
- ❑ African-Caribbean (11)
- ❑ Filipino (12)
- ❑ Latin American, Hispanic, or Latino (13)
- ❑ Arab (14)
- ❑ Southeast Asian (e.g. Cambodian, Malaysian, Laotian) (15)
- ❑ West Asian (e.g. Iranian, Afghan) (16)
- ❑ Korean (17)
- ❑ Japanese (18)
- ❑ Jewish (19)
- ❑ Mediterranean (20)
- ❑ Prefer not to answer (55)
- ❑ Other- Please describe below (88)

D03a. You indicated that your background as "Other". Please describe your background: ____ [conditional if D03 is 'other']

D04. What is the highest level of education you have completed?

- ❑ No formal education (1)
- ❑ Less than grade 9 (2)
- ❑ Some high school / secondary school (3)
- ❑ Completed high school (received secondary school diploma) (4)
- ❑ Some trade or technical training (5)
- ❑ Completed trade or technical training (received certification / diploma) (6)
- ❑ Some college (7)
- ❑ Completed college (received degree or diploma) (8)
- ❑ Some university (9)
- ❑ Completed university (received degree) (10)
- ❑ Post-graduate education (11)

D05. What is your current employment status? *Please check the one that best describes your employment status.**

- ❑ Working for pay, full-time taxable income (30 hours or more per week) (1)
- ❑ Working for pay, part time taxable income (less than 30 hours per week) (2)
- ❑ Working for pay, part time taxable income (less than 30 hours per week, but wish to work full-time) (3)
- ❑ Working for pay, full-time (under the table income) (30 hours or more per week) (4)
- ❑ Working for pay, part-time (under the table income) (less than 30 hours per week) (5)
- ❑ Working for pay, part-time (under the table income) (less than 30 hours per week, but wish to work full-time) (6)
- ❑ Volunteering (working without pay) (7)
- ❑ Student (either part-time or full-time) (8)
- ❑ Retired (9)
- ❑ I am retired but also earning some part-time income (10)
- ❑ On disability (11)
- ❑ Unemployed / not working, but seeking work (12)
- ❑ Unemployed / not working, not seeking work (13)
- ❑ Other – Please describe below (14)

D05a. You indicated "Other" as your current employment status. Please describe your current employment status: ____ [conditional if D05 is 'other']

**Question modified from the employment status item in the Employment Change and Health Outcomes (ECHO) study Rueda. S. et al.*

D06. What is your average personal GROSS yearly income before tax?

- ❑ Less than $10,000 (1)
- ❑ $10,000 to less than $20,000 (2)
- ❑ $20,000 to less than $30,000 (3)
- ❑ $30,000 to less than $40,000 (4)
- ❑ $40,000 to less than $50,000 (5)
- ❑ $50,000 to less than $60,000 (6)
- ❑ $60,000 to less than $70,000 (7)
- ❑ $70,000 to less than $80,000 (8)
- ❑ $80,000 to less than $100,000 (9)
- ❑ $100,000 or more (10)
- ❑ Prefer not to answer (55)

D07. Please check the option that **best** describes your primary source of personal income for this year.

- ❑ Full-time employment (1)
- ❑ Part-time employment (2)
- ❑ Self-employment (3)
- ❑ Disability pension (e.g. Ontario (or other provincial) Disability Support Program, Employment Support and Income Assistance, etc.) (4)
- ❑ Worker’s compensation (5)
- ❑ Employment insurance / Long-Term Disability (LTD) (6)
- ❑ Provincial social assistance program (e.g. Ontario Works, BC Employment & Assistance Program [BCEA], Manitoba Employment and Income Assistance Program [EIA], Manitoba Works, Nova Scotia Employment Support and Income Assistance [ESIA]) (7)
- ❑ Private pension (8)
- ❑ Canada Pension Plan (CPP) – Disability (9)
- ❑ Canada Pension Plan (CPP) – Retirement (10)
- ❑ Informal street-related work such as pan handling (11)
- ❑ Under the table work (12)
- ❑ Student loan (13)
- ❑ Old Age Security (14)
- ❑ Other- Please describe below (88)

D07a. Please describe the “Other” source of your personal income for this year: ____ [conditional if D07 is ‘other’]

D08. What extended medical insurance benefits do you have (to cover costs not paid through provincial health care)?

- ❑ No medical insurance benefits other than provincial health care (0)
- ❑ Benefits through a provincial social assistance program (1)
- ❑ Individual plan (2)
- ❑ Group plan through work (3)
- ❑ Family’s, Parent’s or Partner’s plan (4)
- ❑ Other – Please describe below (5)

D08a. Please describe the “Other” extended medical insurance benefits that you have: ____ [conditional if D08 is ‘other’]

D09. Please check the option that **best** describes what kind of housing you have right now.

- ❑ Single detached house – own (1)
- ❑ Single detached house – rent (2)
- ❑ Townhouse –own (3)
- ❑ Townhouse – rent (4)
- ❑ Condominium apartment – own (5)
- ❑ Condominium apartment – rent (6)
- ❑ Apartment (7)
- ❑ Hotel room (8)
- ❑ Rooming house (9)
- ❑ Shelter (10)
- ❑ Hostel (11)
- ❑ Halfway house (12)
- ❑ Recovery house (13)
- ❑ Prison (14)
- ❑ No fixed address (15)
- ❑ Couch surfing with friends / family (16)
- ❑ On the street (17)

D10. Have you ever been in prison?

- ❑ Yes (1)
- ❑ No (0)

D11. Over the past six months, did you inject any drugs for non-medicinal purposes?

- ❑ Yes (1)
- ❑ No (0)
- ❑ Unsure (66)
- ❑ Prefer not to answer (55)

D12. Have you ever been paid for sex?

- ❑ Yes (1)
- ❑ No (0)
- ❑ Unsure (66)
- ❑ Prefer not to answer (55)

D13. What is your most recent CD4 count? ___________

D14. What was/is your lowest CD4 count ever recorded? _______

D15. What is your most recent viral load? _________________

Just a few last questions about how you heard about and completed the survey:

D16. I heard about the survey from….

- ❑ An email or eblast (1)
- ❑ Poster or recruitment card (2)
- ❑ Presentation about the survey (3)
- ❑ In person - word of mouth (4)
- ❑ Social media (5) – Please describe below
- ❑ Other – Please describe below (6)

D16a. You indicated that you found out about this survey from a "Social media" source. Please describe: _____ [conditional if D16 is 'social media']

D16b. You indicated that you found out about this survey from an "Other" source. Please describe: _____ [conditional if D16 is 'other']

D17. Did you receive any assistance to help you complete this questionnaire?

- ❑ Yes (1)
- ❑ No (0)

D18. What type of device did you complete this survey?

- ❑ Personal Computer (desktop or laptop) (1)
- ❑ Computer at community organization (desktop or laptop) (2)
- ❑ Tablet (iPad, Blackberry Playbook, HTC Tablet) (3)
- ❑ Smart Phone (iPhone, Android) (4)
- ❑ Other (5)

D18a. You indicated that you completed this survey on an "Other" device. Please describe: _____ [conditional if D18 is 'other']

D19. This brings us to the end of the *HIV, Health and Rehabilitation Survey*. Is there anything else you would like to say about the health-related challenges you experience living with HIV or any other health condition, or the rehabilitation services and living strategies that you use to deal with these health-related challenges? If so, please comment below…..

SUBMIT (button)

HIV, Health and Rehabilitation Survey

Thank you for your participation in the *HIV, Health and Rehabilitation Survey*.
If you have any questions, please contact the Research Coordinator at the University of Toronto [Email]; [Phone]
